# Unraveling novel TF-miRNA regulatory crosstalk in metastasis of Soft Tissue Sarcoma

**DOI:** 10.1038/srep09742

**Published:** 2015-05-18

**Authors:** Devyani Samantarrai, Mousumi Sahu, Jyoti Roy, Bedanta Ballav Mohanty, Garima Singh, Chandra Bhushan, Bibekanand Mallick

**Affiliations:** 1 RNAi and Functional Genomics Laboratory, Department of Life Science, National Institute of Technology, Rourkela, India

## Abstract

Cancer metastasis is a disease of extreme clinical relevance, as it is responsible for more than 90% of cancer-associated mortality. The molecular mechanism and critical regulators involved in this complex multi-stage process of metastasis is poorly deciphered in soft tissue sarcomas (STS), a heterogeneous group of rare tumors with high metastatic potential. Therefore, we aimed at identifying miRNA and transcription factor (TF) regulatory networks and paths in STS metastasis. We integrated mRNA and miRNA expression profiles with curated regulations (TF→gene, TF→miRNA, miRNA→gene) from different databases and constructed a potentially active regulatory sub-network in STS metastasis. From functional and topological analysis, we found nine novel regulators of Notch signaling sub-network which are conjectured to play critical role in metastasis of STS. This illustrated that the sub-network is promising for identification of critical regulators. Further analysis deploying our developed tool ‘RiNAcyc’ and computing coverage ratio of known STS associated genes and miRNAs identified a 15 node active path. This potential path highlights the crucial role of BMP2, hsa-miR-24, AP2 and MYC as the up-stream regulators of the path and hsa-miR-215 and TYMS as potential indicator of chemotherapeutic benefit in STS metastasis.

Soft tissue sarcomas (STS) are rare form of cancer comprising of a heterogeneous group of more than 50 histological subtypes derived from mesenchymal tissues^1^. STS range in their behavior from low grade tumors which have the propensity to recur locally to aggressive high grade tumors with the ability to metastasize to distant sites^2^. The rarity of these tumors, poor prognosis, limited effective therapeutic options and ability to metastasize early make them a challenging area of research. The stage of diagnosis and extent of metastasis are the two strongest predictor of patient survival which augments the need for understanding pathogenesis of STS metastasis to facilitate the development of new diagnostic markers and therapeutic targets.

Metastasis is a complex multi-stage process of extreme clinical relevance which is responsible for more than 90% of cancer-associated mortality^3^. This includes escape of tumor cells from primary site, local invasion, entry into local vascular or lymphatic vessels (intravasation), survival in the circulation that includes aggregation with platelets, attachment at distant site by interaction with distant endothelial cells, extravasation, colonization at distant sites and expansion. The molecular mechanism underlying metastatic cascade in STS (non-epithelial cancers) is largely unknown owing to the complexity and heterogeneity of the malignancy as compared to carcinomas (epithelial cancers). The regulators modulating different stages of metastasis of STS are still unidentified making advanced research on STS metastasis an important area of study. Many attempts have been made to predict metastasis in STSs mostly by correlating gene expression patterns with metastatic potential of high grade STS^4^. The prognostic molecular signatures in 89 pleomorphic STS and 30 leiomyosarcomas, a type of STS was independently deciphered by Francis and Lee respectively^5^,^6^. These signatures consisted of more than 200 genes, but did not provide any clear clue towards any emerging biological pathways contributing to metastasis. Chibon and his colleagues identified a set of 67 genes termed as complexity index in sarcomas (CINSARC) which are predicted to be involved in mitosis and chromosome integrity and can predict metastatic outcomes in STS^4^. However, the signatures from these independent studies share little overlap and provide inadequate knowledge about the mechanism of STS metastasis.

Transcriptional and post-transcriptional regulations are the critical components of tumor progression and metastasis which have increasingly garnered the attention of cancer investigators in recent years. The deregulation of transcription factors (TFs), the major regulators controlling expression of different sets of RNAs at transcriptional level, whereas miRNAs mainly acting at post-transcriptional level modulate target mRNA expression influencing multiple steps of the metastatic cascade. miRNAs are known to be involved in a wide array of biological processes including cell differentiation, development, cell death, homeostasis, and fine-tuning their regulation^7^ and their aberrant expression have been shown to be strongly correlated to STS pathogenesis^8^. miRNAs are believed to have genetic switch mechanisms whereby these essentially modulate the target genes expression by regulating TF and other TF-mediated events and vice versa^9^. Thus, a comprehensive coordinated regulatory network for studying complex diseases demands integration of both transcriptional and post transcriptional regulation.

Gene expression profile has been utilized largely for identification of underlying mechanism of a disease through interactome and network studies. To identify and understand the signaling and regulatory interactions that are operational in a system due to gene expression changes, the concept of active sub-network (ASN) was devised^10^. A similar approach was adopted for finding deregulated ASN in a regulatory network by Backes et al^11^. A novel methodology, termed as CASNet (*C*onsistent *A*ctive *S*ub*net*works) was developed later by Gaire et al. to extract ASN by integrating difference in gene expression^12^. Moreover, various studies have been done to uncover the miRNA-TF regulatory networks in different cancers such as glioblastoma, osteosarcoma^13^,^14^. Majority of these studies were focused mainly on feed forward loop (FFL) motif and generated networks from the computationally predicted regulations of miRNAs and TFs. The extrapolation and identification of regulatory pathways from these ASNs will be icing on the cake and can throw more light on the key factors and their regulators responsible for a disease. To this end, Jiang and his group^15^ used the breadth first search algorithm to identify active regulatory pathways from ASNs in alzheimer's disease. The algorithm traversed all vertices and only provided the pathways, but did not perform any kind of enrichment study for a list of genes/miRNAs which is essential to show their significant associations with a biological process or system.

In this study, we contemplated differentially expressed (DE) miRNAs and mRNAs of different STS types for metastasis analysis taking into consideration of the fact that most of the human cancer types share small number of cellular, molecular and biochemical traits which form the basis of hallmarks of cancers. We amalgamated expression profiles of different STSs, curated data on various regulatory interactions (TF→gene, TF→miRNA, miRNA→gene) from different databases to identify potentially active sub-network (PASN) from a larger bio-molecular network which might be active during metastasis of STS. Integrating these with known STS related genes and miRNAs to improve prediction accuracy followed by network topology and functional studies, we were able to find potentially significant STS metastasis specific paths, hubs and regulators from the network. The curated regulations considered in our study to generate ASNs and paths are more discerning as compared to predicted regulations adopted in other methods to identify key modulators. Furthermore, we developed a standalone application called RiNAcyc (RNA-interacting Nodes in Acyclic paths) for identification of all acyclic paths from a network which would be enriched with DE genes/miRNAs and will provide coverage ratio of a list of entities that are known STS associated genes/miRNAs in our study. This tool available at our website, http://vvekslab.in/tools.html can be exploited to perform similar studies in other disease systems. In addition to finding acylic paths, we generated a sub-network from the components of a core pathway, the Notch signaling pathway and identified novel regulatory components which might participate in STS metastasis-specific Notch signaling. This signaling is more alluring for investigation as this highly conserved pathway is reported to be involved in developmental processes, proliferation, apoptosis and play critical role in modulating other oncogenic signaling besides its role in sarcoma invasion and metastasis^16^,^17^,^18^. The outline of the approach adopted is provided in Fig. 1.

Thus, our study provided a pool of genes and miRNAs with an unexplored potential to be critical regulators during STS metastasis progression. This miRNA-TF regulatory network generation and path analysis, first of its kind in STS will provide a framework for further understanding and targeting crucial step in STS progression to harness these for therapeutics.

## Results

### DE mRNAs & miRNAs in STS

Microarray analysis of the two data sets using GeneSpring GX 12.6 (Agilent Technologies) resulted in 343 common DE mRNAs and 250 common DE miRNAs in STS, with p < 0.05 and fold change ≥2.0. Functional analysis of the DE genes and miRNAs using Metacore ™ resulted in 47 genes and 11 miRNAs to be involved in metastasis of cancers.

### Identification of active TF-miRNA sub-network and functional analysis

A curated TF-miRNA regulatory network was constructed using Cytoscape by integrating curated data from databases (refer to Materials and Methods). The generated network included 300 miRNAs, 414 TFs and 2470 target genes that formed 3184 nodes and 6153 interactions/regulations (Fig. 2A). A STS metastasis specific potential active TF-miRNA regulatory sub-network was obtained by mapping the 47 DE genes and 11 miRNAs having role in metastasis as active seed nodes. Of these, only 21 genes and 2 miRNAs mapped to the TF-miRNA regulatory network as seed nodes. These DE nodes were further connected to their immediate neighbors irrespective of directions to generate a PASN (Fig. 2B). The sub-network consisted of 55 TFs, 120 target genes and 64 miRNAs and 483 pairs of regulations. The significance of these potential active TF-miRNA co-regulatory sub-network in STS metastasis was assayed using hypergeometric test. For hypergeometric test, following values were considered-the curated TF-miRNA regulatory network of 3184 nodes of which 158 were known to be STS associated, the PASN of 239 nodes of which 36 are known STS associated. As a result, the elements in the active sub-network significantly enriched the set of known STS-associated genes and miRNAs (P = 3.12 × 10^−10^).

To have a general view of this regulatory active sub network, we calculated degrees (connectivity) and their distributions, which are basic topological network measures. In this network, the degree values of the genes, miRNAs and TFs ranged from 1 to 18, 1 to 80 and 1 to 78 respectively. The degree distribution for genes, miRNAs and TFs were found to be strongly right-skewed, indicating that most nodes had a low degree, while only a small portion of nodes had a high degree called as hubs (Fig. S1).

We screened nodes with greater than 10 degrees as potential hubs which are assessed for their potential roles in STS metastasis (Table 1). We obtained 2 miRNA hubs (hsa-miR-373 and hsa-miR-21), 10 TF hubs (MYC, FOS, PTEN, ESR1, SP1, TP53, EGR1, RELA, YY1, CREB1) and 1 target gene hub (VEGFA). Of these thirteen hubs (Fig. S2), six (hsa-miR-373, MYC, PTEN, ESR1, TP53 and VEGFA) are reported to be involved in STS pathogenesis and metastasis^14^,^19^,^20^,^21^.

Functional analysis of active sub-network for enriched pathways yielded many curated pathways which might be active in STS metastasis (Table 2). From 239 genes and miRNAs of active sub-networks, 175 genes were found to be enriched in 16 pathways (p < 0.001) (Table 2) as obtained from analysis in consensus path database (CPDB) and Metacore. Remaining 64 miRNAs were analyzed using DIANA-mirPath^22^ that yielded 38 pathways including Notch signaling.

### Identification of novel components of Notch signaling pathway in STS metastasis

From CPDB pathway analysis, we found 5 genes (YY1, RELA, STAT3, TCF3, LEF1) from the active sub-network to be part of Notch signaling. We generated a sub-network from these 5 genes by mapping them as seed nodes to the active sub-network involved in STS metastasis and extracted the network they formed with their neighboring nodes (Fig. 3). We obtained a STS metastasis specific Notch signaling network with 26 nodes and 50 edges (interactions). This consists of 6 target genes, 11 TFs and 9 miRNAs. Further, network topological analysis for identifying significant critical regulators resulted in 7 TFs hubs and 2 miRNAs hubs (Fig. S3). From these hub regulators, three are implicated in STS pathogenesis and rest are found to be involved in other mesenchymal malignancies. Thus, these 9 regulatory hub molecules might play important roles in Notch signaling mediated STS metastasis pathogenesis. Their role and association with Notch is described in Table 3.

### Identification of potential active TF-miRNA regulatory paths in STS metastasis

From the potentially active TF-miRNA regulatory sub-network in STS metastasis, we extracted all the acyclic pathways from zero-indegree to zero-outdegree using RiNAcyc tool developed by us. 1565 acyclic paths with 3 or more nodes which could be potentially active paths in STS metastasis were obtained. These paths consisted of 127 genes and miRNAs. The maximum length of path we obtained was 14. To identify the significant active paths from these potential active paths, we mapped the known STS genes and miRNAs obtained from Malacard, Human MicroRNA Disease Database (HMDD) database and Metacore. 221 STS associated genes and 16 miRNAs were obtained which were associated with STS pathogenesis. Of these 237 STS associated genes and miRNAs, 27 were found to be present in 1565 potential paths. We then calculated the coverage ratio as described in materials and methods section for each path to measure the probability of occurrence of the path in STS metastasis. We obtained 12 paths with p < 0.001 (calculated using hypergeometric test) that were considered significantly active TF-miRNA regulatory paths in STS metastasis (Table 4). Due to some common components among these significant paths, we combined them to form a single significant path (Fig. 4).

We also carried out topology measure/degree connectivity of the nodes (Table S1) forming the significant active paths that consisted of 5 TF hubs (MYC, FOS, PTEN, ESR1, TP53). This shows the critical regulators from the network to be playing important role in STS metastasis by being part of the active significant path. Three of the TF hubs (MYC, FOS, TP53) also formed part of STS metastasis-specific Notch signaling networks.

The 12 significant paths obtained are inferred to be potentially involved in STS metastasis. Firstly, while correlating the extent to which the components of all significant active paths appeared in the set of known STS associated genes and miRNAs, we obtained 27 STS associated entities from the list of 127 genes/miRNAs forming 1565 potential active regulatory paths. Further, the 12 active paths consisted of 15 genes/miRNAs of which 9 were reported to be associated with STS. Using hypergeometric test, it was seen that entities in all of the active pathways significantly enriched (p = 0.00053) the set of known STS-associated genes and miRNAs. Secondly, Metacore™ analysis was performed for remaining 6 genes/miRNAs (FOS, hsa-miR-19a, hsa-miR-22, hsa-miR-24, hsa-miR-215, hsa-miR-302c) where all were found to be related to STS indirectly by their involvement in other mesenchymal tumors/sarcomas except hsa-miR-302c.

## Discussions

De-regulation at the transcriptional and post-transcriptional level has a crucial impact on the oncogenesis of STS. In this study, we established a TF-miRNA regulatory network which might be playing a critical role in the muti-stage process of metastasis of STS at both transcripts and protein level. There is vast literature dedicated to the study of carcinomas, but not much information available on critical regulators of the metastatic pathway in STS. Elucidating the regulators controlling metastasis is vital for improving patient outcome and developing novel therapeutics. Therefore, we were impelled to construct an active TF-miRNA regulatory network in STS metastasis with the help of curated regulations and deduced critical regulators, novel notch signaling components and significant active paths which have illuminated the most significant regulatory nodes in metastatic cascades operating in STS. This work represents the first TF-miRNA regulatory network in STS.

We obtained 16 pathways from the functional analysis of the active sub-network analysis. Among them, a number of pathways have role reported in metastasis of cancers, such as TGFβ receptor, EGFR1, androgen receptor, Notch signaling pathways and few more. We selected Notch signaling to assess its role in STS metastasis because of its reported crosstalks with other oncogenic signaling pathways like EGFR, NF-κB, Akt, Sonic hedgehog (Shh), mammalian target of rapamycin (mTOR), Ras, Wnt, platelet-derived growth factor (PDGF), TGFβ and androgen receptor signaling which pave tumor aggressiveness^23^,^24^,^25^. Our Metacore analysis also revealed crosstalks among members of Notch pathway and members of other critical pathways like TGFβ and EGFR1. The notch pathway appears to be critical in the invasion and metastasis of sarcomas as well and ideally placed to regulate other signaling pathways involved in tumorigenesis.

The STS metastasis specific Notch signaling network analysed consisted of 28 nodes. Of these, 2 TFs (TP53, MYC), 3 miRNAs (hsa-miR-373, hsa-miR-34a, and hsa-miR-31) and 1 target gene (IL6) are reported to be associated with STS pathogenesis^14^,^19^,^20^,^26^. Further network topological analysis revealed nine novel components for this pathway which might also be active in Notch signaling during STS metastasis. These novel regulatory molecules include MYC, TP53, STAT3, RELA, YY1, FOS, SP1, hsa-miR-21 and hsa-miR-34a. Their role and association with Notch signaling is briefed in Table 3. Majority of the regulators were seen to play important roles in osteosarcoma and thus show their potential role in being part of Notch signaling pathway in metastasis of STS. Further validation of the regulators is warranted for establishing this hypothesis.

The most interesting part of the study is the deduction of acyclic paths from the potential active TF-miRNA regulatory network. We obtained 12 statistically significant paths (p < 0.001) which when integrated formed a 15 node path composed of different TFs, miRNAs and target genes. This composite path might be active in STS metastasis and plays a crucial role in the disease progression. All the regulators of the paths except miR-302c have considerable evidences to support their direct involvement in sarcoma (osteosarcoma, rhabdomyosarcoma, Kaposi sarcoma). However, hsa-miR-302c has been observed to act as a tumor suppressor in hepatocellular carcinoma by inhibiting angiogenesis, an important event in metastasis^27^. Hence, miR-302c can be assumed to play an important tumor suppressive role in regulation of crucial step in STS metastasis. We also found some other regulatory interactions in the path which could be relevant to STS metastasis, such as hsa-miR-24→MYC. hsa-miR-24 has been found to be down-regulated in osteosarcoma which is acting as an inhibitor of cell proliferation^28^. It is also seen to target cell cycle regulator MYC and inhibit proliferation in hematopoietic cell differentiation^29^. Proliferation being the initial crucial step of metastasis, we hypothesize that hsa-miR-24 might play a tumor suppressive role during STS metastasis. Its role in invasion and metastasis of carcinomas is also well defined^30^. MYC oncogene then might mediate miRNA mediated down-regulation of other genes in STS metastasis as indicated from our active path (Fig. 4) (MYC→hsa-miR-221, MYC→hsa-miR-26a). Role of hsa-miR-221 in osteosarcoma was recently elucidated where it was reported to induce malignant phenotype by inhibiting PTEN (hsa-miR-221→PTEN)^31^. The PTEN regulating miRNA, hsa-miR-26a (hsa-miR-26a→PTEN) is seen to be up-regulated in liposarcoma^32^ indicating it might play an oncogenic role in mesenchymal cancer or STS. The function of PTEN in regulating oncomiR (hsa-miR-19a) and onco-suppressor miRNAs (hsa-miR-302c, hsa-miR-22) in malignant transformation and metastasis have been reported earlier, and thus can be predicted to have potential role in STS metastasis as well. hsa-miR-19a belongs to the cluster miR-17-92 and is found to be higher in metastatic subline of osteosarcoma cell SAOS-2^33^. Guled et al identified expression pattern of miRNAs including hsa-miR-22 from a series of miRNA profiling of leiomyosarcoma and undifferentiated pleomorphic sarcoma and linked them to targets known to be involved in Leiomyosarcoma (LMS) development and progression^34^. hsa-miR-22 has also been seen to have a inhibitory role in clonogenic and anchorage-independent cell growth of some mesenchymal malignancies even at modest over-expression levels. Anchorage independent growth is an important criterion for initiating metastasis and hsa-miR-22 might act as a tumor suppressor in STS as well.

Comparative genomic hybridization (CGH) studies on copy number in dedifferentiated and pleomorphic liposarcomas have revealed gain in copy number of oncogenes, notably ESR1 (Estrogen receptor 1), a receptor in estrogen signaling^21^. Owing to the important role of this signaling in proliferation and differentiation, ESR1 can be assumed to play a role in STS metastasis by mediating its effect on FOS, the proto-oncogene which has been implicated as regulators of cell proliferation, differentiation, transformation and metastasis. Additionally, clinical studies have revealed that more than half cases of human osteosarcomas analyzed are c-fos positive and are associated with high relapse frequency and poor chemotherapeutic response.

Tumors included in STS are relatively more chemotherapeutic resistant than other sarcomas^35^. Therefore, it is important to identify regulators modulating chemotherapeutic sensitivity and resistance. We observed that all of the 12 significant paths we identified end with TP53→hsa-miR-215→TYMS. Thymidylate synthase (TYMS) catalyzes dUMP→dTMP and is the sole intracellular *de novo* source of thymidylate, an essential precursor for DNA biosynthesis and a target of the most widely used chemotherapeutic agents like TDX against gastrointestinal malignancies^36^. hsa-miR-215 on the other hand has been seen to play an important role in tumor chemoresistance in osteosarcoma^37^. Song and his colleagues showed that although hsa-miR-215 directly targeted TYMS, chemoresistivity towards TDX was solely due to hsa-miR-215 mediated down-regulation of denticleless gene (DTL) and not because of TYMS. They demonstrated that miR-215 suppressed DTL, the destabilizing factor of TP53 to promote TP53 stabilization and subsequently inhibit cell growth and cause cell cycle arrest in the G2 phase. J. Ju then independently suggested the presence of a positive feedback loop between hsa-miR-215 and TP53 mediated via down-regulation of DTL^36^. Our microarray analysis showed up-regulation of both DTL and TYMS around 7 folds and down-regulation of hsa-miR-215 around 11 folds in all STS sub-types. From the above supporting evidences, we can predict a positive feedback regulation between TP53 and TYMS occurring in STS metastasis. TYMS has also been implicated in metastasis of carcinomas^38^. Overall, we can hypothesize hsa-miR-215 and TYMS as potential candidate for prognostic indicator of chemotherapeutic benefits in STS metastasis. This can be further investigated to elucidate TYMS up-regulation mediated metastasis and chemosensitivity via hsa-miR-215 regulation.

From the predicted regulatory cascades of 15 nodes (Fig. 4), it was observed that MYC regulation mediated by either BMP-2 via hsa-miR-24 or AP2 TF formed the up-stream molecules of all pathways which might play a crucial role in STS metastasis progression. The activation of BMP2 and AP2 TF might cause the induction of cascade effect and can lead to de-regulated expression of the down-stream molecules and their targets. The role of these molecules in oncogenesis and metastasis in other sarcomas has been reported^39^,^40^. Moreover, hsa-miR-24 has an unexplored potential of being a tumor suppressor in STS metastasis which can further modulate the regulatory cascade. As described above, our predicted path indicates that MYC regulates its targets in STS metastasis via regulating different miRNAs, thus making MYC a potential candidate for targeted therapies.

Our method of uncovering paths can be implemented to delineate critical regulatory cascades in other disease systems to unveil the complex TF-miRNA-gene regulatory relationships. However, further validation by biological experiments of the potential regulations is warranted for confirming their role in other diseases and more specifically in metastasis of STS of this study. Even though our study provides a comprehensive landscape of TF-miRNA regulatory network in STS metastasis, recent reports about novel cross-talks among the diverse RNA species like mRNAs and other non-coding RNAs indicate that there remain more dynamic regulations to be considered. One such promising intricacy involves interplay among competing endogenous RNAs (ceRNAs) and target RNAs, wherein ceRNAs regulate them by competing for shared miRNA response elements (MREs). Incorporating this novel crosstalk among the varied classes of RNAs in generating regulatory network will lead to significant gain in understanding of their role in development of STS and other cancers.

## Methods

### Gene and miRNA expression profile of high grade STS

Human STSs are a heterogeneous group of aggressive mesenchymal cancers where metastasis related death occurs in 50% of the patients with high grade STS. Despite their heterogeneous nature, these must be sharing common features in their metastatic process. Therefore, we performed a comprehensive analysis on five major subtypes of STS for gene expression studies and six different sub-types of STS for miRNA expression studies to obtain a common set of DE genes and miRNAs. We retrieved primary high grade STS gene expression profile (GSE21122) of Affymetrix's platform (Human Genome U133A technology) and miRNA expression profile (GSE36982) of Illumina platform from Gene expression omnibus database (GEO)^41^. The mRNA microarray dataset comprised of 158 samples including 9 normal fat as control and 149 samples of five major sub-types of STSs (Leiomyosarcoma, myxoid/round-cell liposarcoma, dediffentiated liposarcoma, myxofibrosarcoma and pleomorphic liposarcoma). For miRNA expression analysis, we considered 58 samples of six different STS subtypes (Leiomyosarcoma, pleomorphic liposarcomas, myxoid liposarcoma, dediffentiated liposarcoma, myxofibrosarcoma, malignant fibrous histiocytoma and pleomorphic liposarcoma) and 2 normal fat samples as control. Both the data sets were analyzed using GeneSpring GX 12.6 software (Agilent technologies). For gene expression analysis, pre-processing of data is performed by going for gene level expression analysis in GeneSpring wherein if multiple probe sets corresponded to the same gene, then the expression values of these probe sets were averaged. The robust multi-chip averaging algorithm was used for summarization with subsequent baseline normalization of the log-summarized values for each probe set. The data were then filtered to remove probe sets with lowest 20 percentile of all intensity values. For miRNA expression analysis, data was normalised using quantile normalization and filtered using flags. The subsequent analysis steps were same for both mRNA and miRNA, wherein the data were subjected to ANOVA analysis, incorporating the Benjamini-Hochberg FDR multiple testing correction with p-value < 0.05. The probe sets were further filtered on the basis of fold change cut-off of 2.0, a standard criterion for differential expression to obtain significantly DE genes and miRNAs^13^. The probe sets for each expression value were then mapped to Entrez gene ID for further analysis.

### Functional analysis of DE genes and miRNAs

Functional analysis of DE mRNAs and miRNAs obtained from microarray data processing was carried out to check for their involvement in different biological processes using Metacore™ (Thomson Reuters). The DE genes and miRNAs with established role in the multi-stage process of metastasis of cancer were screened out for further analysis.

### Genes and miRNAs related to STS

The genes and miRNAs associated with STS pathogenesis were collected from two different databases. Genes were collected from the STS specific annotations in MalaCards human disease database version 1.05^42^. MalaCards mines and merges forty four data sources to generate information about 16,919 diseases. miRNAs associated with STS were obtained from HMDD^43^. This database contains manually curated and experimentally validated miRNAs de-regulated in diseases from published studies. Due to less number of miRNAs obtained from this database, we additionally mined Metacore™ (Thomson Reuters) to obtain more deregulated miRNAs in STS. A gene or miRNA is considered to be involved in STS if it is reported in any one of the sub-types of sarcoma considered in our study.

### Curated regulatory relationships and network generations

**Figure 1:**
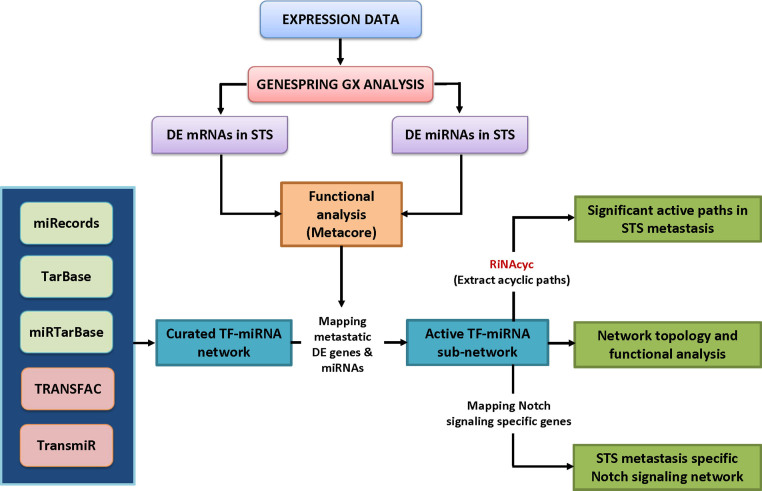
Overview of the approach adopted.

**Figure 2:**
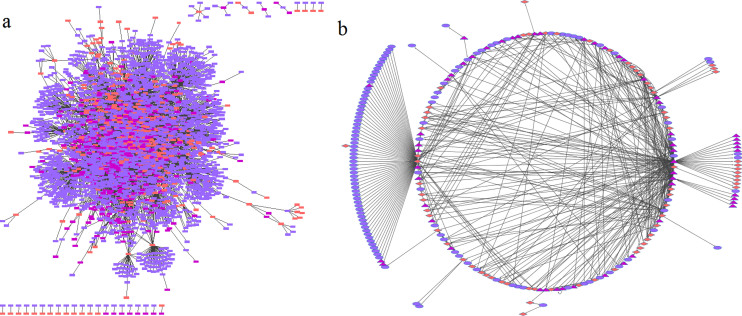
(A) Curated TF-miRNA regulatory network. (B) Active sub-network in STS metastasis. 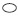 Target genes △ Transcription factors ⋄ miRNAs → Interaction between TF and target ⊣ Inhibition of target by miRNAs.

**Figure 3:**
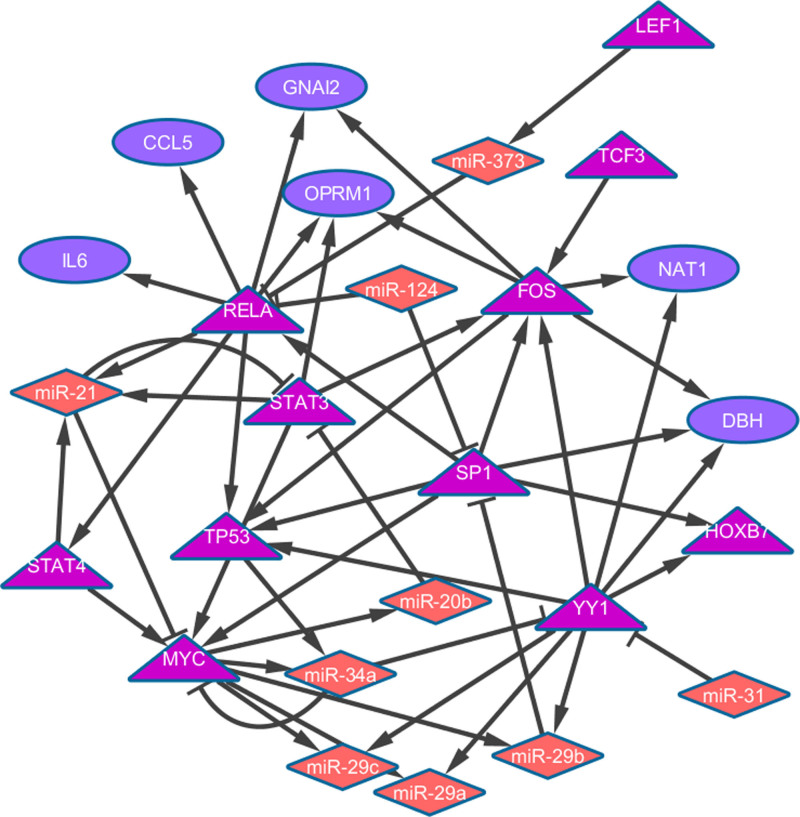
STS metastasis specific Notch signaling network. 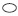
 Target genes △ Transcription factors ⋄ miRNAs → Interaction between TF and target ⊣ Inhibition of target by miRNAs.

**Figure 4:**
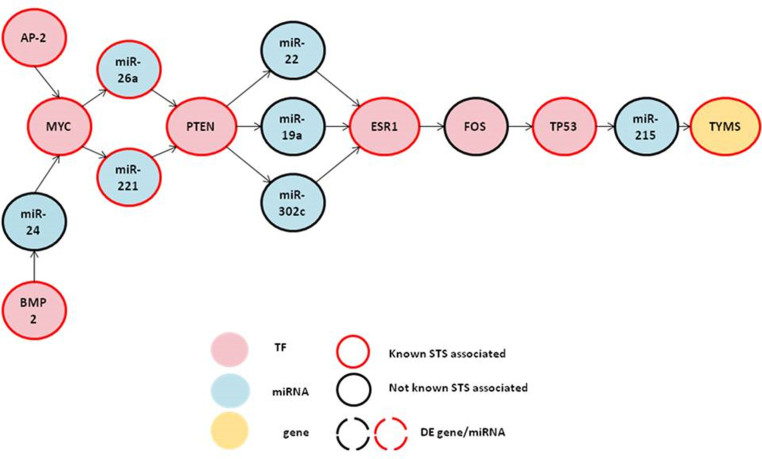
Association of 12 significant active TF-miRNA regulatory paths in STS metastasis.

The regulatory relationships among TFs, miRNAs and target genes (TF→gene, TF→miRNA, miRNA→genes) were obtained from five different curated databases: TransmiR (version 1.2)^44^, TRANSFAC (version 11.4), miRecords (version 3)^45^, miRTarbase (release 2.5)^46^ and TarBase (version 5.0)^47^. In our analysis, the term “gene” includes both TF genes and non-TF genes and the term “gene targets” includes only non-TF genes. All TFs and genes obtained from these databases were mapped to their Entrez gene ID to maintain uniformity and remove redundancy associated with gene name aliases. The miRNA-gene regulations from three databases- miRecords, miRTarbase and TarBase were merged to obtain one uniform non redundant set of miRNA-mRNA relationships. With these above mentioned curated regulatory relationships, we generated a curated TF-miRNA regulatory network using Cytoscape 3.1.1^48^. The redundant edges of the regulatory networks generated were collapsed into one and all the self directed loops were pruned from the network. An overview of the approach is given in Fig. 1.

### TF-miRNA sub-network generation and analysis

A potentially active TF-miRNA sub-network in STS metastasis was generated by mapping entities which are the common DE genes and miRNAs with a role in metastasis obtained from Metacore™ analysis, as active seed nodes. Any neighboring undirected node connected to the active seed node was extracted from the network to form a potential active TF-miRNA sub-network in STS metastasis.

With an aim of finding critical regulatory elements, we performed network topology and functional analysis by ignoring direction of the nodes. The PASN in STS metastasis was analysed considering degree (connectivity), the basic topological measure and their distribution to assess the network characteristics. We determined the degrees of nodes and degree distribution of our network to identify hubs (nodes having large number of connections) in the network which are likely to play important regulatory roles.

We used NetPath of CPDB, a manually curated pathway resource in humans to perform gene set enrichment analysis and identify signal transduction pathways overrepresented with genes involved in STS metastasis-specific regulatory networks^49^. These enriched pathways were further interpreted using Metacore™ (Thomson Reuters). For finding pathways associated with miRNAs, we used DIANA-mirPath, a web based computation tool^22^. It takes into account the combinatorial effect of co-expressed miRNAs in modulating a pathway. It embeds DIANA-microT, Pictar, or TargetScan to predict the targets of miRNAs of interest and finds KEGG pathway annotations enriched in the target. Further, from the pathways obtained, we selected Notch signaling pathway for sub-network generation and network topology and functional analysis because of its importance in being involved with a array of critical signaling pathways and role in metastasis.

### Identification and evaluation of paths from TF-miRNA regulatory sub-network

In our study, paths means a series of connected molecules (TFs, miRNAs, target genes) of a minimum length of 3 nodes and maximum of 30, which represents a signaling cascade occurring in a cell. The paths which connected multiple TFs, miRNAs and target mRNAs were identified from the active sub-network to uncover the regulatory cascades involved in transcriptional and post transcriptional regulations playing important role in STS metastasis pathogenesis. For identification of active paths from the active sub-network, all the acyclic paths with zero in-degree to zero out-degree nodes were identified using RiNAcyc, the tool developed by our group. It is a freely available standalone tool written in python using DFS algorithm that can be implemented in any disease system for generating acyclic paths. We selected paths enriched with 50% DE genes and miRNAs and evaluated to identify the significant potential active regulatory paths operating in STS metastasis. Evaluation was based on finding the coverage ratio of known STS genes and miRNAs for each path. Coverage ratio is defined as the probability of path occurring in STS; higher the coverage ratio of a path, higher its chances of occurring in STS metastasis. It was calculated as number of known genes/miRNAs in STS divided by path length (total number of genes/miRNAs in the path). The statistical significance was calculated using hypergeometric test by the tool.

## Author Contributions

B.M. and D.S. conceived the idea. D.S., M.S. and B.M. performed the experiment and data analysis. B.B.M. and J.R. assisted in analysis and fine-tuning the interpretations. C.B. developed the RiNAcyc. D.S., B.M. and J.R. have written the manuscript. All authors reviewed and edited the manuscript. B.M. supervised the project.

## Additional Information

**How to cite this article**: Samantarrai, D. *et al.* Unraveling novel TF-miRNA regulatory crosstalk in metastasis of Soft Tissue Sarcoma. *Sci. Rep.* 5, 9742; DOI:10.1038/srep09742 (2015).

## Supplementary Material

Supplementary Information Supplementary Figures & Tables

## Figures and Tables

**Table 1:** Nodes acting as hubs in potential active sub-network

Nodes	In-degree	Out-degree	Total degree
**hsa-miR-373**	4	76	80
**MYC**	32	46	78
**FOS**	22	19	41
**PTEN**	17	9	26
**ESR1**	9	15	24
**SP1**	2	22	24
**VEGFA**	18	0	18
**TP53**	10	5	15
**hsa-miR-21**	5	8	13
**EGR1**	3	8	11
**RELA**	3	7	10
**YY1**	2	8	10
**CREB1**	1	9	10

**Table 2:** Enriched curated pathways from the potential active TF-miRNA sub network in STS metastasis

Pathway	Members	p-value
**IL2**	STAT4; STAT3; STAT1; CREB1; ELK1; IL2; EIF4E; RELA; TERT; YBX1; ATF2	2.15E−09
**Oncostatin M**	ERBB2; STAT3; STAT1; FOS; EGR1; TP53; RELA	3.66E−07
**BCR**	STAT3; STAT1; FOS; CREB1; CTNNB1; ELK1; CDK4; HNRNPK; RELA; GTF2I; ATF2	5.70E−07
**ID**	MYOD1; TCF3; TCF7L2; ELK3; ELK1; ELK4	6.30E−07
**TGF-beta Receptor**	TP53; FOS; SP1; CDC25A; ESR1; CTNNB1; CDK4; CD44; LEF1; MYC; CTCF; ATF2	1.58E−06
**TSLP**	EIF4E; RELA; STAT4; STAT3; STAT1	4.85E−06
**Gastrin**	STAT3; SP1; CREB1; CTNNB1; IL2; RELA	1.06E−05
**Leptin**	ERBB2; STAT3; STAT1; ESR1; SP1; CFL2; EIF4E	1.11E−05
**Androgen Receptor**	CTNNB1; STAT3; ESR1; SP1; CDC25A; CREB1; AHR; PTEN; ATF2	7.24E−05
**Prolactin**	ERBB2; STAT3; STAT1; FOS; ESR1; RELA	0.000229
**IL6**	ERBB2; STAT3; STAT1; FOS; IL6; EIF4E	0.000311
**IL5**	CTNNB1; ELK1; STAT3; STAT1; ATF2	0.000312
**Notch**	YY1; RELA; STAT3; LEF1; TCF3	0.000755
**IL4**	ELK1; STAT3; CXCR4; STAT1; ATF2	0.001102
**Ghrelin**	RELA; ELK1; GNAI2; CREB1	0.001356
**IL11**	RELA; STAT3; STAT1	0.002799
**EGFR1**	ERBB2; STAT3; STAT1; ENO1; SP1; FOS; CREB1; ADAM9; PTEN; ELK1; TP53; RELA; MYC	0.004329
**CRH**	RELA; GNAI2; CREB1	0.009277

**Table 3:** STS metastasis-specific Notch signaling network hubs

Nodes	Out-degree	In-degree	Total degree	Evidences in support of their association with Notch signaling	References
**MYC**	5	5	10	Direct target of Notch1; amplified in STS with a potential role in sarcomagenesis	[50]
**RELA**	6	4	10	Regulated by Notch signaling in cancers	[51–52]
**YY1**	8	2	10	Association with high molecular weight Notch complex; over-expressed in human osteosarcomas	[53–54]
**FOS**	5	4	9	Encoded by Notch pathway target gene and modulates cell fate in cancer cells	[55]
**SP1**	6	2	8	important for Notch1 transcription; a key regulator in miRNA-TF regulatory network in osteosarcoma proliferation	[13]
**STAT3**	4	2	6	Notch signaling activates STAT3 pathway via notch effector HES1 TF; involved in survival and proliferation of osteosarcoma cells	[56]
**TP53**	1	4	5	Notch signaling is an important functional player in TP53 suppression pathways during tumor progression	[57]
**hsa-miR-21**	2	3	5	Correlation in expression of hsa-miR-21 and Notch1; involved in osteosarcoma cell invasion and migration as an oncogene	[58–59]
**hsa-miR-34a**	2	2	4	A potential regulator of Notch signaling; involved in osteosarcoma cell invasion and migration as a tumor suppressor	[60]

**Table 4:** Significant active TF-miRNA regulatory paths in STS metastasis

Most significant paths	Known STS	Path length	p-value
AP2→MYC→hsa-miR-26a→PTEN→hsa-miR-22 →ESR1→ FOS→TP53→ hsa-miR-215→TYMS	7	10	0.0007
AP2→MYC→hsa-miR-26a→PTEN→hsa-miR-19a →ESR1→FOS→TP53 →hsa-miR-215→TYMS	7	10	0.0007
AP2→MYC→hsa-miR-26a→PTEN→hsa-miR-302c →ESR1→FOS→TP53 →hsa-miR-215→TYMS	7	10	0.0007
AP2→MYC→hsa-miR-221→PTEN→hsa-miR-22 →ESR1→FOS→TP53→ hsa-miR-215→TYMS	7	10	0.0007
AP2→MYC→hsa-miR-221→PTEN→hsa-miR-19a →ESR1→FOS→TP53 →hsa-miR-215→TYMS	7	10	0.0007
AP2→MYC→hsa-miR-221→PTEN→hsa-miR-302c →ESR1→FOS→TP53 →hsa-miR-215→TYMS	7	10	0.0007
BMP-2→hsa-miR-24→MYC→hsa-miR-26a→PTEN→hsa-miR-22 → ESR1→TP53→hsa-miR-215→TYMS	7	10	0.0007
BMP-2→hsa-miR-24→MYC→hsa-miR-26a→PTEN→hsa-miR-19a → ESR1→TP53→hsa-miR-215→TYMS	7	10	0.0007
BMP-2→hsa-miR-24→MYC→hsa-miR-26a→PTEN→hsa-miR-302c → ESR1→TP53→hsa-miR-215→TYMS	7	10	0.0007
BMP-2→hsa-miR-24→MYC→hsa-miR-221→PTEN→hsa-miR-22 → ESR1→TP53→hsa-miR-215→TYMS	7	10	0.0007
BMP-2→hsa-miR-24→MYC→hsa-miR-221→PTEN→hsa-miR-19a → ESR1→TP53→hsa-miR-215→TYMS	7	10	0.0007
BMP-2→hsa-miR-24→MYC→hsa-miR-221→PTEN→hsa-miR-302c → ESR1→TP53→hsa-miR-215→TYMS	7	10	0.0007
