# Supplementary material for: Unraveling novel TF-miRNA regulatory crosstalk in metastasis of Soft Tissue Sarcoma
Source: Sci Rep. 2015 May 18;5:9742. doi: 10.1038/srep09742 (PMC4434893; doi:10.1038/srep09742)
Supplement: Supplementary Information — Supplementary Figures & Tables [file srep09742-s1.doc]

**Supplementary File**

**Unraveling novel TF-miRNA regulatory crosstalks in metastasis of Soft Tissue Sarcoma**

Devyani Samantarrai, Mousumi Sahu, Jyoti Roy, Bedanta Ballav Mohanty, Garima Singh, Chandra Bhushan and Bibekanand Mallick^*^

RNAi and Functional Genomics Laboratory, Department of Life Science,

National Institute of Technology, Rourkela, India

**
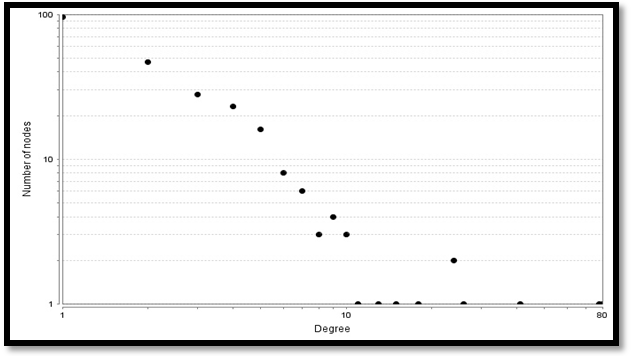
**

**Figure S1: Degree distribution of active sub-network in STS metastasis**


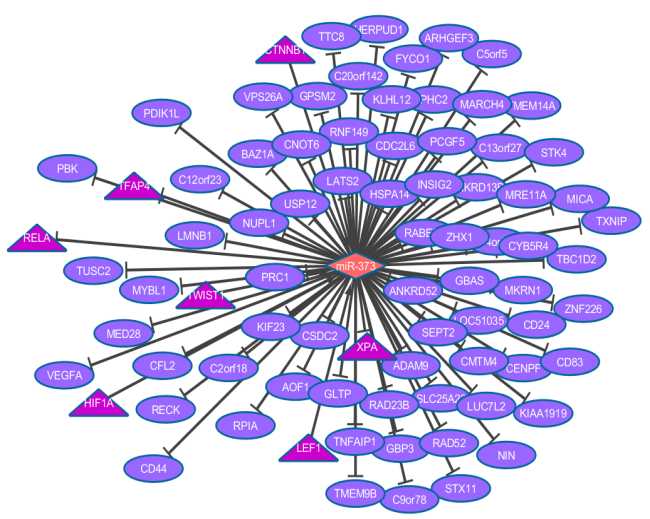

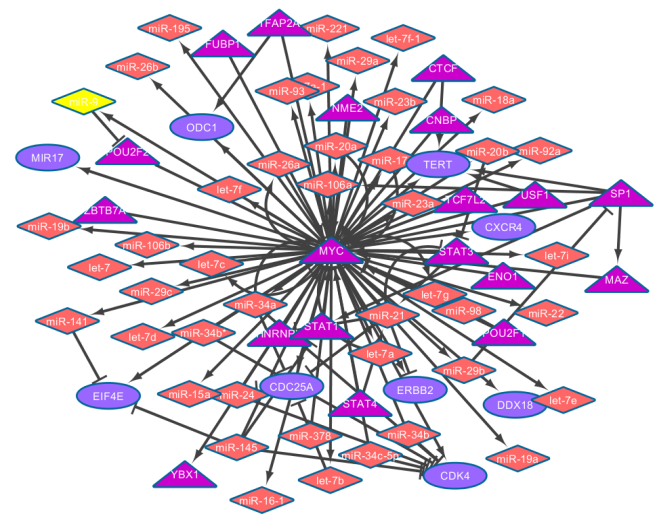

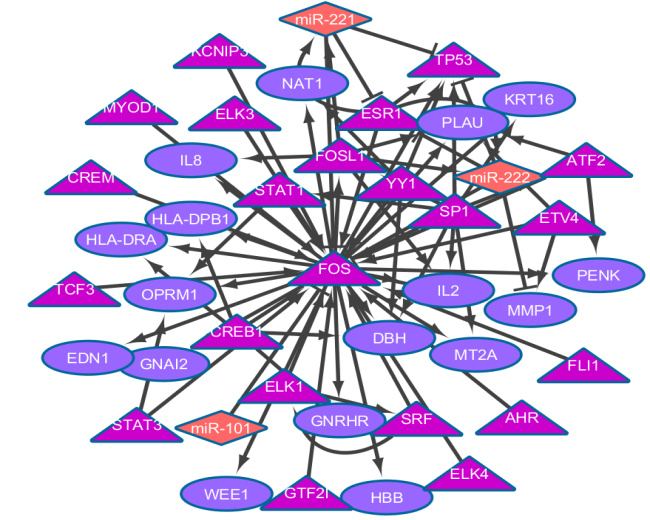

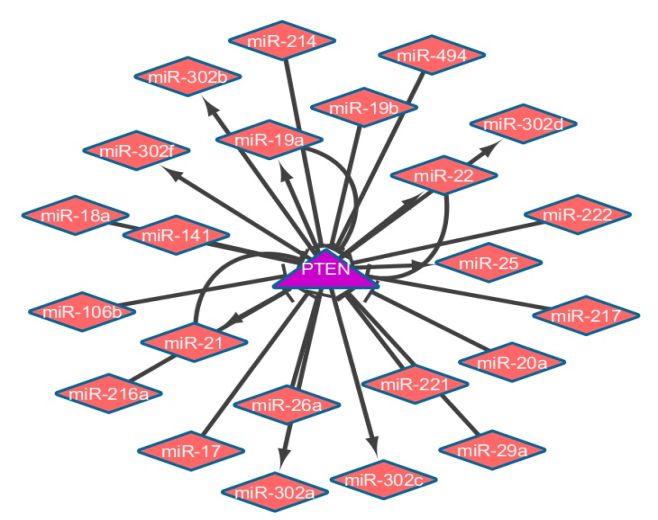


**A**

**B**

**C**

**D**


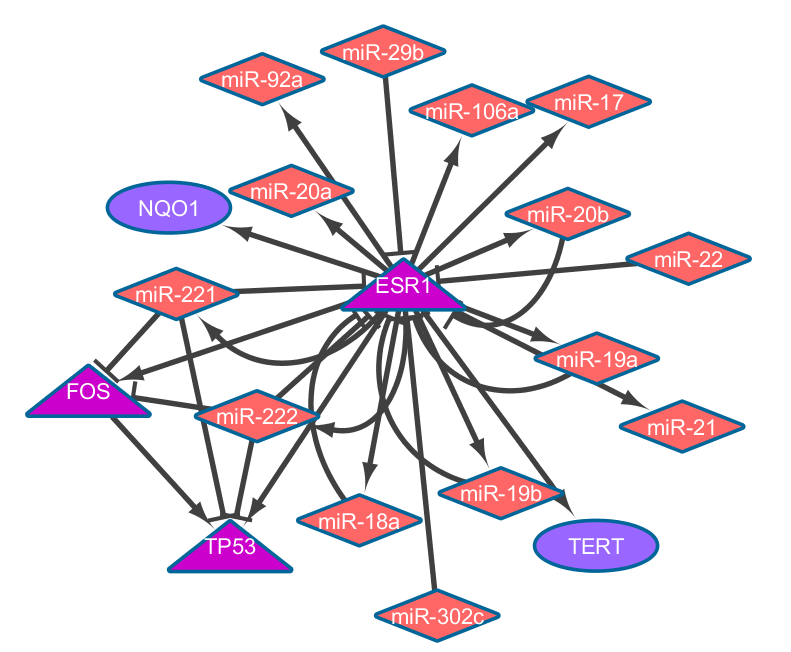

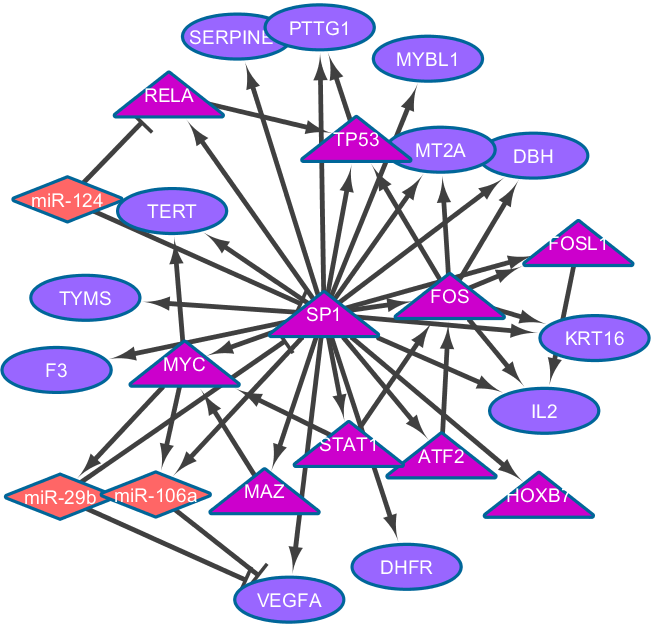


**E**

**F**


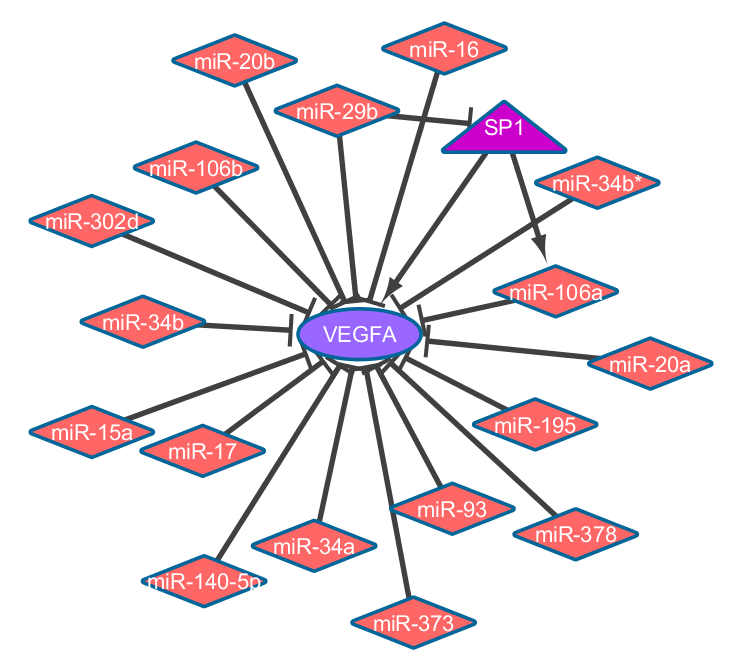

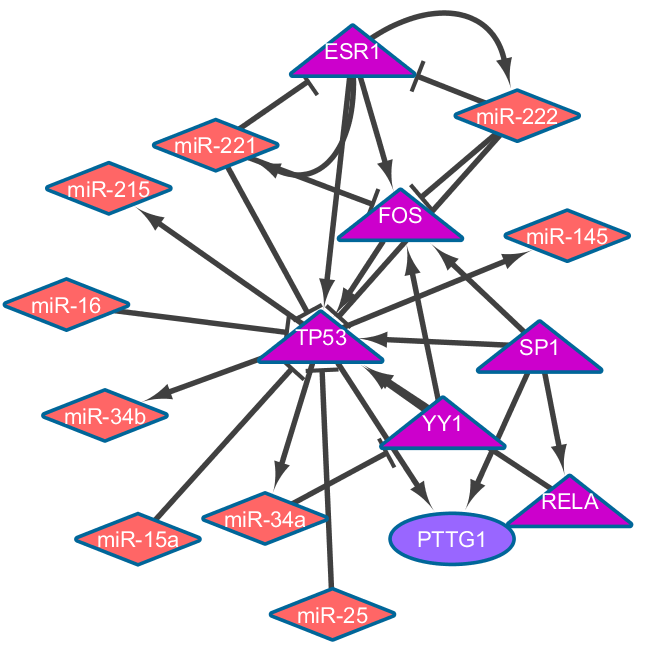


**G**

**H**


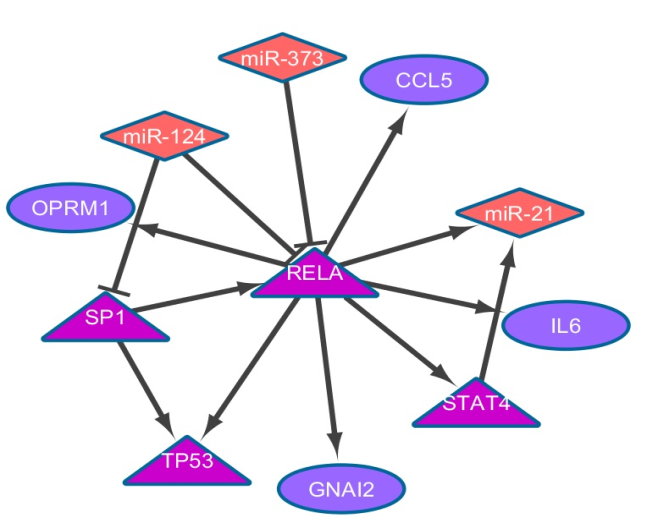

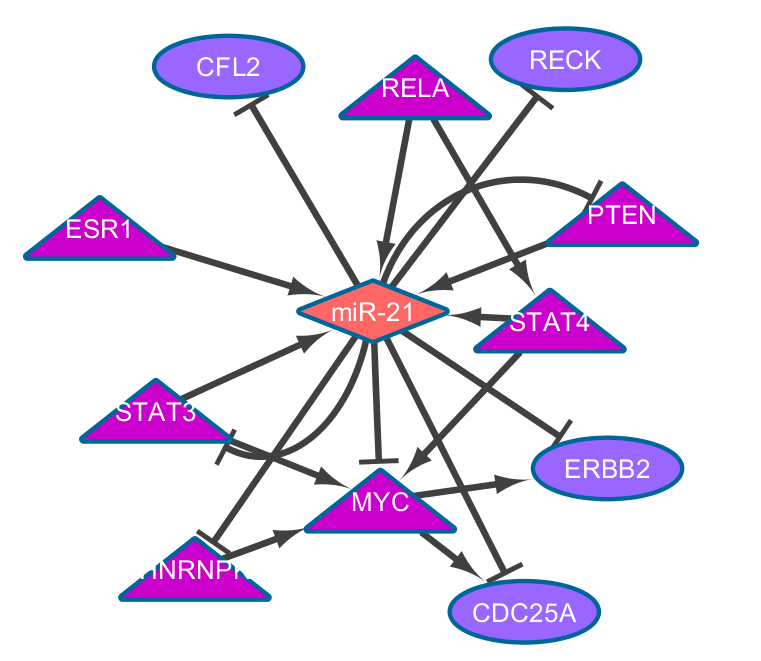


**J**

**I**


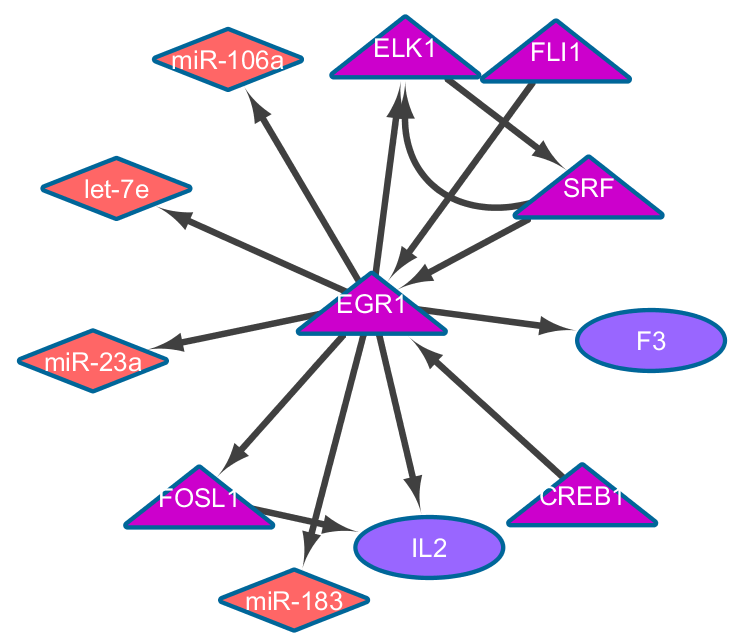

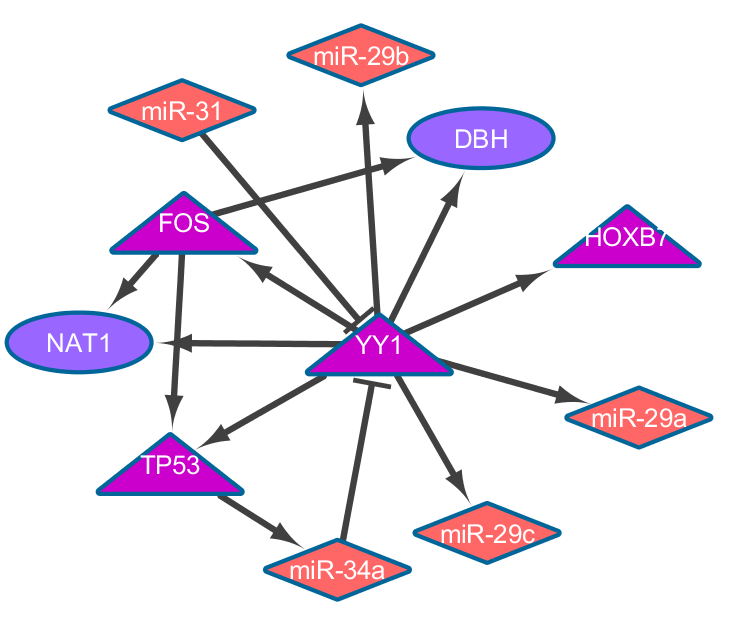

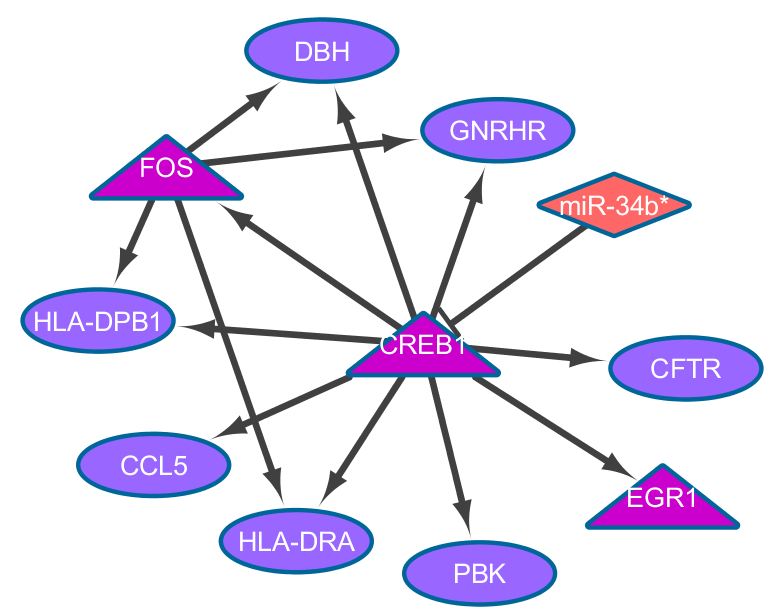


**L**

**K**

**M**

**Figure S2: 13 hubs in STS metastasis specific potential active TF-miRNA regulatory sub-network**. A. hsa-miR-373, B. MYC, C. FOS, D. PTEN, E. SP1, F. ESR1, G. VEGFA, H. TP53, I. RELA, J. hsa-miR-21, K. EGR1, L. YY1, M. CREB1


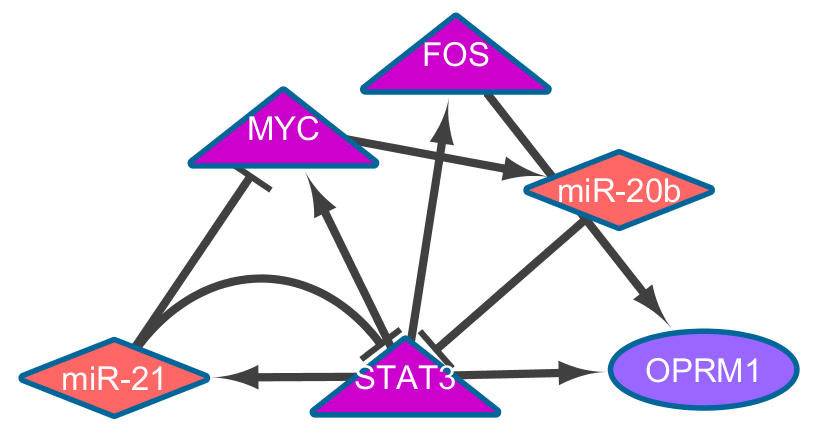

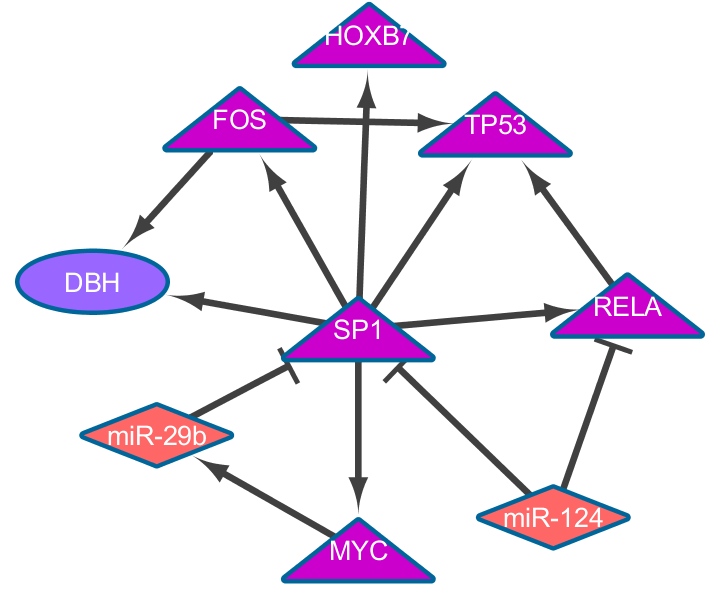

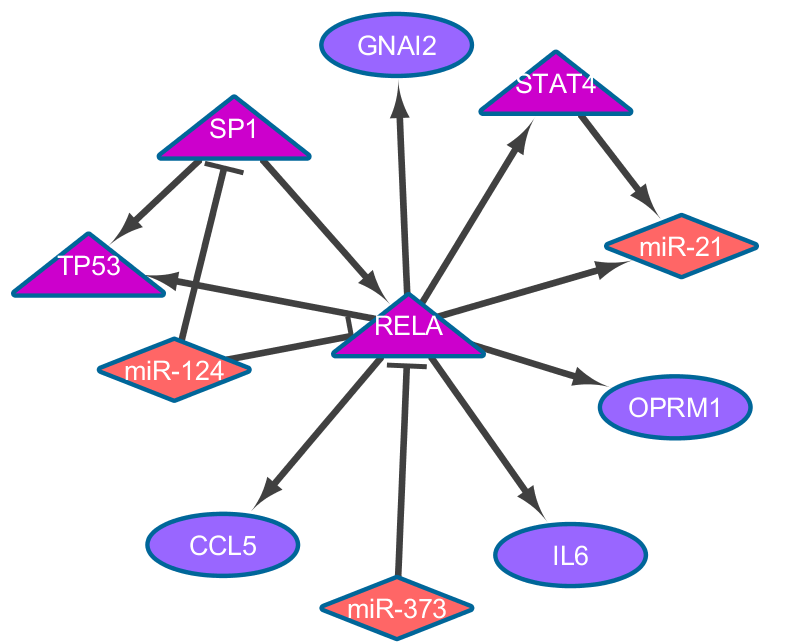

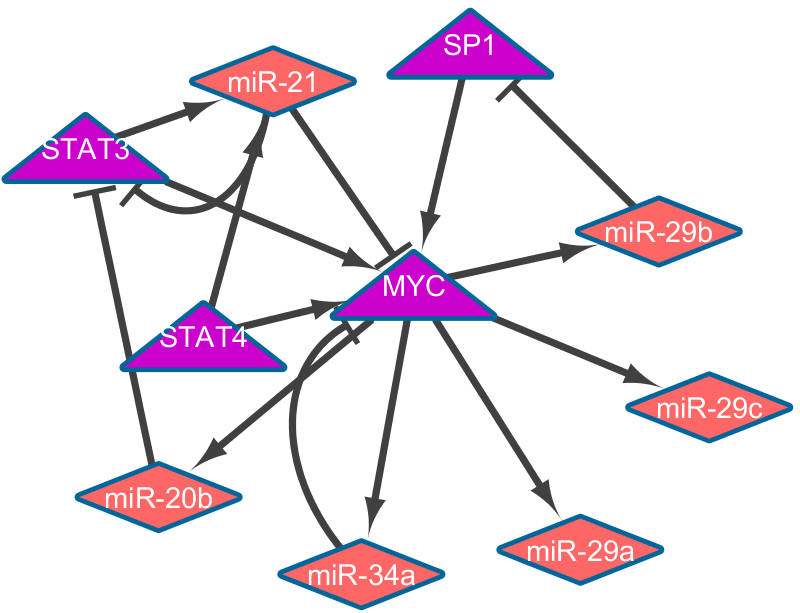

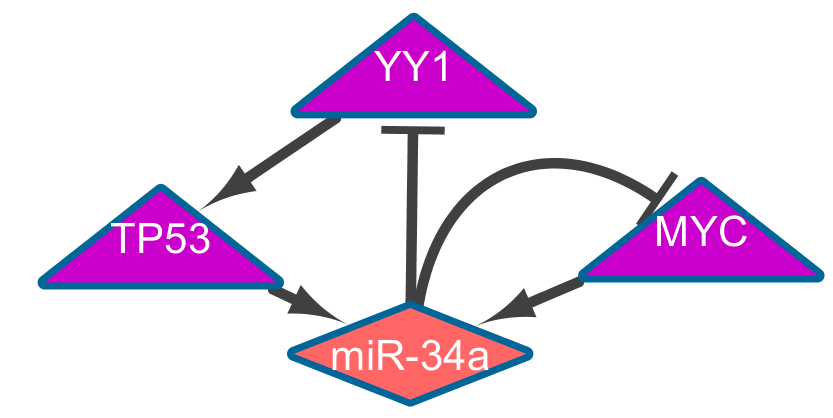

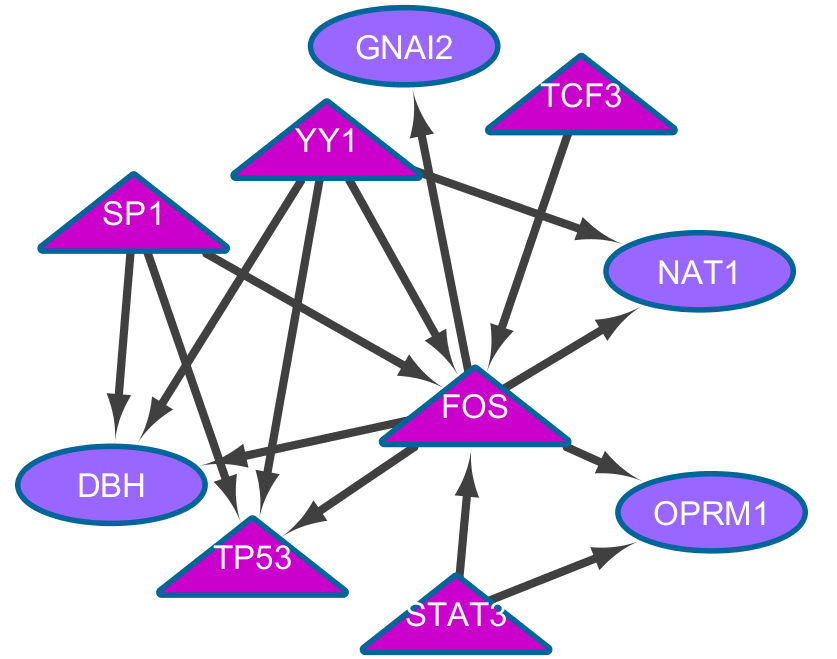

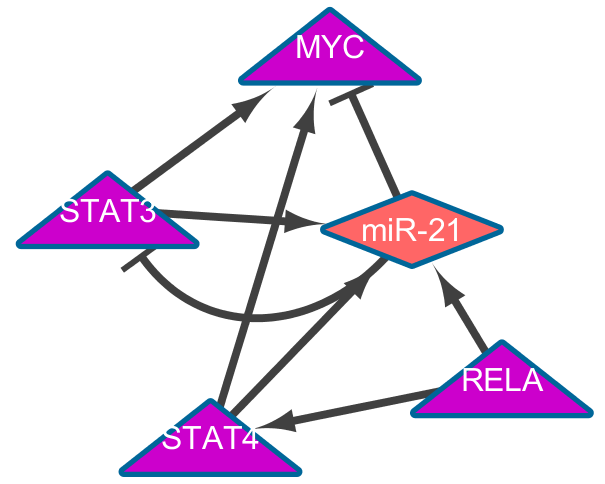

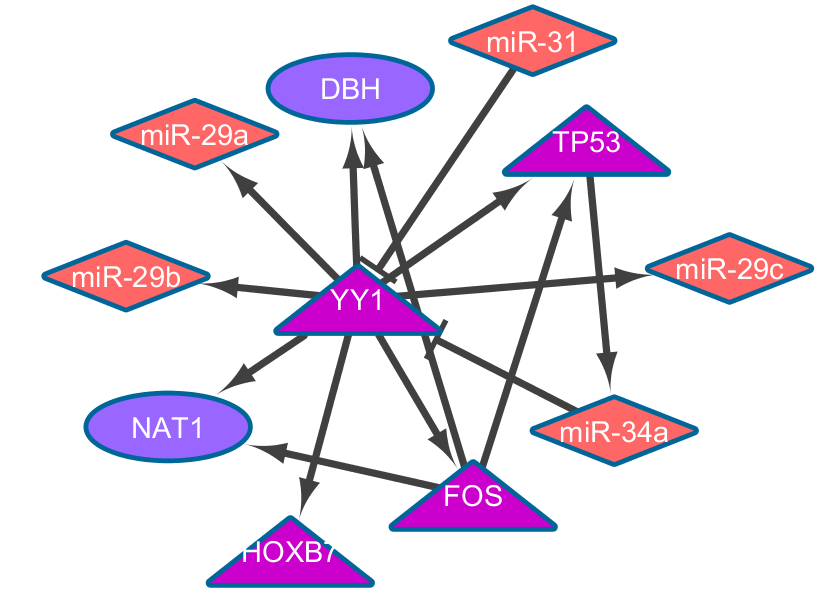


**A**

**B**

**C**

**D**


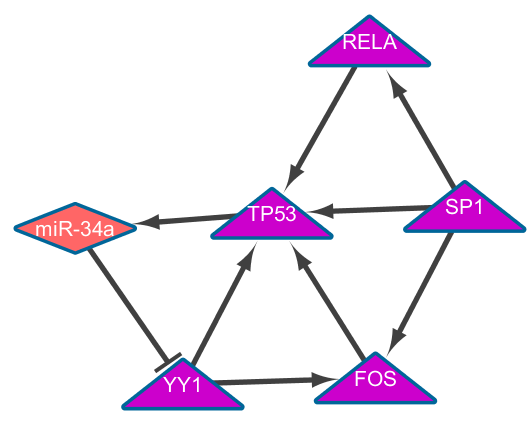


**I**

**E**

**F**

**G**

**H**

**Figure S3: 9 hubs in STS metastasis specific Notch signaling sub-network.**

A. STAT3, B. SP1, C. RELA, D. MYC, E. hsa-miR-34a, F. FOS, G. hsa-miR-21, H. YY1, I. TP53

**Table S1: D**egree connectivity of the 15 genes/miRNAs forming the active path

**G**

**H**

| **Nodes** | **Edge count** | **In-degree** | **Out-degree** |
| --- | --- | --- | --- |
| **MYC** | 75 | 32 | 43 |
| **FOS** | 41 | 22 | 19 |
| **PTEN** | 26 | 17 | 9 |
| **ESR1** | 24 | 9 | 15 |
| **TP53** | 16 | 10 | 6 |
| **hsa-miR-221** | 7 | 3 | 4 |
| **hsa-miR-24** | 6 | 3 | 3 |
| **hsa-miR-19a** | 5 | 3 | 2 |
| **hsa-miR-215** | 5 | 1 | 4 |
| **TFAP2A (AP2)** | 5 | 0 | 5 |
| **hsa-miR-22** | 4 | 2 | 2 |
| **hsa-miR-26a** | 4 | 1 | 3 |
| **TYMS** | 3 | 3 | 0 |
| **BMP2** | 2 | 0 | 2 |
| **hsa-miR-302c** | 2 | 1 | 1 |
